# Cairns Aitken, CBE, MD, FRCPE, FRCPsych

**DOI:** 10.1192/bjb.2018.78

**Published:** 2019-04

**Authors:** Bruce Ritson

**Emeritus Professor of Rehabilitation Studies, Edinburgh University Medical School, UK**


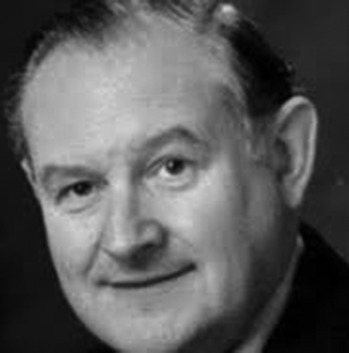


Cairns Aitken, who died of pulmonary fibrosis on 12 March 2018 at age 84, had a distinguished career as a Scottish psychiatrist and had many prominent roles in management within Scottish universities and the health service. Perhaps his major achievement was when, as Chair of the Royal Infirmary Trust, he masterminded the move of the Royal Infirmary of Edinburgh from its historic site in the city centre to its new building, on the periphery of the city, in Little France. This involved negotiating the rebuilding contract via the private finance initiative. He recognised the importance of clinicians' involvement in management at a time when many were wary of its inevitable conflicts and disputes. When Cairns encountered a challenge, he would always face it head-on and not shrink from difficult or unpopular decisions; he would identify a goal and then form a project team of skilled experts around him who would all work together to overcome obstacles and succeed.

Cairns held a number of other major positions. From 1984 to 1987 he was Chairman of the British Broadcasting Corporation Scottish Medical Advisory Board, and from 1991 to 1996 he was a member of the General Medical Council. He was appointed to the Human Genetics Advisory Commission in 1996. This public body was created to report on new developments in human genetics, and the consequences thereof, in relation to public health, insurance, patents and employment, and to advise on ways to build public confidence in – and understanding of – these developments.

In addition to all of Cairns’ clinical and academic work, he became Chairman of the Council of Napier College and helped steer it from being a local college to becoming a national polytechnic and finally the renowned university it is today. This transition was an enormous achievement involving hours of painstaking negotiations. In 1990 he was made a Fellow of Napier University.

Many would have felt such an achievement was sufficient but Cairns then threw himself into a series of challenging projects, first as Vice Dean and then as Dean of the Faculty of Medicine from 1988 to 1991. Finally, he was appointed Vice Principal of Edinburgh University and was responsible for planning and budgeting at what was an exceptionally challenging time. He thus played a major part in forming the foundations of the University as it is today. He was a director of the Lothian Health Board from 1991 to 1993, which led to his role in the re-siting of the Royal Infirmary.

Cairns was born on 20 December 1933, an only child. His father was a qualified accountant, but his parents ran a hotel in Dunoon. Cairns attended Dunoon Grammar School before going to board, first at Cargilfield in Edinburgh and then at Sedbergh School in Yorkshire. He went on to study medicine at Glasgow University. While he was a medical student his mother died of severe asthma. He vividly recalled the stress and worry caused when she had severe breathless attacks. This had a profound effect on him and he felt the experience influenced his career choice and clinical outlook throughout his life.

Early exposure to psychiatry came from a chance meeting with a fellow medical student who told Cairns about free lodgings if you helped as a student at the Gartnavel Royal Mental Hospital. He described how, for 2 years, he enjoyed free accommodation, meals and an introduction to the fascinating challenges of a traditional psychiatric hospital.

Qualifying as a doctor in 1957, he went as an exchange Fellow to McGill University in Montreal. He obtained a short-service commission in the Royal Air Force (1959–1962) and joined the Institute of Aviation Medicine in Farnborough. This enabled him to travel widely and develop his clinical and research interest in treating phobias of flying. During this time, he had the fascinating experience of visiting NASA in its very early days. He was part of a delegation to Washington to hear the first American astronaut, John Glenn, give his report on his return from space to President Kennedy and the nation.

Cairns completed his specialist training in psychiatry at the Maudsley Hospital in London. In 1966 he was headhunted by Professor Carstairs to return to Scotland, first as a lecturer and then senior lecturer in the department of psychiatry, and then as a consultant psychiatrist at the Royal Edinburgh Hospital. Here he continued his research into asthma and psychosomatic medicine. In 1974 he was appointed professor of rehabilitation studies in Edinburgh. This new position was designed for someone who combined an interest in disability with an understanding of the interplay of biological, psychological and social factors in motivating or frustrating recovery. To meet these objectives, Cairns recognised the importance of a team including not only nurses and medical staff but also psychologists, physiotherapists, occupational therapists, the patients themselves and their families. This biopsychosocial approach to healthcare, for which he had advocated for so long, became a practical reality.

He edited the *Journal of Psychosomatic Research* from 1979 to 1986, was President of the Society for Research in Rehabilitation from 1981 to 1983 and was President of the International College of Psychosomatic Medicine from 1989 to 1994. He was a member of the Council for Professions Supplementary to Medicine from 1983 to 1990.

After so many years of public service, Cairns was awarded a CBE in 1998. Among his other honours, he received the Order of Merit of the Polish Republic in recognition of the important and continuing links between the Edinburgh and Polish medical schools.

In retirement he pursued many interests, for example, visiting and documenting all the inhabited islands of Scotland, tracing the journeys of Bonnie Prince Charlie and visiting all the Scottish hydroelectric schemes. Each of these projects was approached with energy and scientific rigour. They involved the preparation of meticulous reports and photographs. He was a skilled photographer and many of his photographs are in the Scran collection, available to the public. In his approach to these projects one detected the passionate and meticulous way in which he set himself objectives and approached challenges throughout his career.

Cairns was very fortunate in having a family who provided love, support and infinite tolerance. He was devoted to his wife Audrey and their three children, Robin, Gail and Shona. Tragically, Shona died of a bone tumour while a student at St Andrew's University. Cairns was also very proud of his two grandsons whom he was thrilled to see gain places at Cambridge and at his own, much-loved University of Edinburgh.

This article is based substantially on the article published online by *The Scotsman* on 24 April 2018. https://www.scotsman.com/news/obituaries/obituary-prof-cairns-aitken-psychiatrist-who-played-a-major-role-in-the-creation-of-edinburgh-royal-infirmary-1-4728854.

